# Supplementary material for: Efficient and accurate extraction of in vivo calcium signals from microendoscopic video data
Source: eLife. 2018 Feb 22;7:e28728. doi: 10.7554/eLife.28728 (PMC5871355; doi:10.7554/eLife.28728)
Supplement: Transparent reporting form [file elife-28728-transrepform.pdf]

eLIFE
1st Floor
24 Hills Road
Cambridge CB2 1JP, UK
P 01223 855340
W elifesciences.org
T @elife

eLife's transparent reporting form

We encourage authors to provide detailed information *within their submission* to facilitate the interpretation and replication of experiments. If you have any questions, please contact us: editorial@elifesciences.org.

Sample-size estimation

- You should state whether an appropriate sample size was computed when the study was being designed
- You should state the statistical method of sample size computation and any required assumptions
- If no explicit power analysis was used, you should describe how you decided what sample (replicate) size (number) to use

Please outline where this information can be found within the submission (e.g., page numbers or figure legends), or explain why this information doesn't apply to your submission:

This information doesn't apply to our submission because our submission contains no statistical tests.

Replicates

- You should report how often each experiment was performed
- You should include a definition of biological versus technical replication
- The data obtained should be provided and sufficient information should be provided to indicate the number of independent biological and/or technical replicates
- If you encountered any outliers, you should describe how these were handled
- Criteria for exclusion/inclusion of data should be clearly stated
- High-throughput sequence data should be uploaded before submission, with a private link for reviewers provided (these are available from both GEO and ArrayExpress)

Please outline where this information can be found within the submission (e.g., page numbers or figure legends), or explain why this information doesn't apply to your submission:

In page 24-25, we described our procedure of simulating data and details of processing those data. From page 25 to page 27, we listed the details of collecting experimental data and analyzing those data using CNMF-E and PCA/ICA approaches.

Statistical reporting

- Statistical analysis methods should be described and justified
- Raw data should be presented in figures whenever informative to do so (typically when N per group is less than 10)
- For each experiment, you should identify the statistical tests used, exact values of N, definitions of center, methods of multiple test correction, and dispersion and precision measures (e.g., mean, median, SD, SEM, confidence intervals; and, for the major substantive results, a measure of effect size (e.g., Pearson's r, Cohen's d)
- Report exact p-values wherever possible alongside the summary statistics and 95% confidence intervals. These should be reported for all key questions and not only when the p-value is less than 0.05.

Please outline where this information can be found within the submission (e.g., page numbers or figure legends), or explain why this information doesn't apply to your submission:

All results shown in our work are direct visual comparisons between results using different approaches. The metrics we used for comparing were intuitive and clearly described in the corresponding main context or figure legends. We don't have statistical tests in our manuscript.

(For large datasets, or papers with a very large number of statistical tests, you may upload a single table file with tests, Ns, etc., with reference to page numbers in the manuscript.)

Additional data files ("source data")

- We encourage you to upload relevant additional data files, such as numerical data that are represented as a graph in a figure, or as a summary table
- Where provided, these should be in the most useful format, and they can be uploaded as "Source data" files linked to a main figure or table
- Include model definition files including the full list of parameters used
- Include code used for data analysis (e.g., R, MatLab)
- Avoid stating that data files are "available upon request"

Please indicate the figures or tables for which source data files have been provided:

We packed our MATLAB code for generating **all** figures in the manuscript into one file **code.zip** and named each subfolder as Fig* for easy references. However, it's noteworthy that we didn't always produce figures by running the code from the beginning to the end. In many cases, we just selectively run cells of each *.m file, and some manual interventions are needed.

As for packages of CNMF-E, PCA/ICA and CNMF, they can be freely downloaded from their own GitHub repo. Here are those links: CNMF-E: https://github.com/zhoupc/CNMF_E; PCA/ICA: https://github.com/mukamel-lab/CellSort; CNMF: https://github.com/epnev/ca_source_extraction.

For all simulated data, we can run the code to generate them again. As for experimental data, we uploaded them to https://datadryad.org/. The temporal review link is http://datadryad.org/review?doi=doi:10.5061/dryad.kr17k
